# Family Music

**Published:** 1857-01

**Authors:** 


### FAMILY MUSIC.

MUSIC, like paintings and statuary, refines, and elevates, and sanctifies. Song is the language of gladness, and it is the utterance of devotion. But coming lower down it is physically beneficial; it rouses the circulation, wakes up the bodily energies, and diffuses life and animation all around. Does a lazy man ever sing? We never heard it. Does a milk-and-water character ever strike a stirring note? Never. Song is the outlet of mental and physical activity, and increases both by its exercise. No child has completed a religious education who has not been taught to sing the Songs of Zion. No part of our religious worship is sweeter than this. In David's day, it was a practice and a study. Such papers as Willis's New York Weekly *Musical World*, at $2 a year, and Woodbury's *Musical Pioneer*, monthly, at half-a-dollar a year, abound in valuable articles on Church and Family music, and merit a liberal patronage.

